# Supplementary material for: Biofunctionalized Nanostructured Yttria Modified Non-Invasive Impedometric Biosensor for Efficient Detection of Oral Cancer
Source: Nanomaterials (Basel). 2019 Aug 22;9(9):1190. doi: 10.3390/nano9091190 (PMC6780737; doi:10.3390/nano9091190)
Supplement: Supplementary file 1 [file nanomaterials-09-01190-s001.pdf]

Supporting Information

Biofunctionalized nanostructured yttria modified non-invasive impedometric biosensor for efficient detection of oral cancer

Suveen Kumar[1,2], Shweta Panwar[1], Saurabh Kumar[1], Shine Augustine[1] and Bansi D. Malhotra[1]*

[1]Nanobioelectronics Laboratory, Department of Biotechnology, Delhi Technological University, Delhi-110042, India

[2]Department of Chemistry, University of Delhi, Delhi-110007, India

*Corresponding Author: bansi.malhotra@gmail.com

Instruments: Crystallinity and information about thephase of the synthesized product were obtained using monochromatic X-ray diffractometer) [Bruker D-8 Advance] with Cu-Kα radiation (λ=1.5406 Å). Morphology and particle size were determined using transmission electron microscopy at an accelerating voltage of 200 kV (Tecnai G2 30 U-twin, Tecnai 300 kV ultra twin microscope). The morphology of nY_2O_3, APTES/nY_2O_3/ITO, and anti-CYFRA-21-1/APTES/nY_2O_3/ITO electrodes were investigated using Field emission scanning electron microscopy (FE-SEM). The Fourier transform infrared spectrometer (FT-IR) (Shimadzu, IRAFFINITY) was used to determine the presence of functional groups and bonds present in APTES/nY_2O_3/ITO and anti-CYFRA-21-1/APTES/nY_2O_3/ITO electrodes. The electrochemical response studies were conducted using a three-electrode cell via a Potentiostat (Autolab Netherlands). The fabricated electrode acted as the working electrode, Ag/AgCl as the reference electrode and platinum (Pt) as the counter electrode. ELISA plate reader (iMark, Bio red, USA) was used for determination of CYFRA-21-1 concentration in saliva of oral cancer patients.

Chemicals and Reagents: Yttrium (III) nitrate hexahydrate and 3-aminopropyl triethoxysilane (APTES) were purchased from Alfa Aesar. N-Cetyl-N,N,N-trimethyl ammonium bromide (CTAB) and 1-ethyl-3-(3-dimethylamino) carbodiimide (EDC), N-hydroxysuccinimide (NHS), sodium monophosphate, sodium diphosphatedihydrate, sodium chloride, potassium ferricyanide, potassium ferrocyanide were procured from Fisher Scientific. Urea was purchased from SRL laboratories. All chemicals were of 99±0.5% purified and were used without any further purification. Phosphate buffer saline (PBS) solution of pH 7.0 was prepared using sodium

monophosphate (0.05 mol L^{-1}), sodium diphosphate dihydrate (0.05 mol L^{-1}) and sodium chloride (0.9%). Fresh PBS solution were prepared using Milli-Q water having resistivity of 18 MΩ cm and stored at 4 ˚C prior to use. CYFRA-21-1 and anti-CYFRA-21-1 were purchased from Ray Biotech, Inc., India. These biomolecules were further diluted by using PBS buffer of pH 7.0. ELISA Kit of CYFRA-21-1 was procured from Kinesis DX, USA

2.3. Patient's saliva samples collection and estimation the concentration of CYFRA-21-1: We collected saliva samples of OC patients form Rajiv Gandhi Cancer Institute and Research Centre, Delhi (India). Prior to collection of the saliva samples, we obtained ethical and biosafety approval both from Delhi Technological University, Delhi (R. No. BT/IEBC/2014/714) and Rajiv Gandhi Cancer Institute and Research Centre (R. No. RGCIRC/IRB/60/2014). Written consent was taken from each patient before collection of saliva. First of all, 5 mL of milli Q water was used for rinsing of mouth and expectorated in the sterilized sample collection tube. The collected saliva was centrifuged at 2800 rcf at room temperature for 30 min. Thereafter, supernatant was collected in sterilized sample collection tube and further stored at -20 ˚C till further use

The concentration of CYFRA-21-1 in saliva samples of OC patients was determined through enzyme linked immunosorbent assay (ELISA) technique. In this process anti-CYFRA-21-1 coated microtiter plate was used and followed all the steps as mentioned in CYFRA-21-1 ELISA kit .The measurements were conducted at 450 nm on ELISA plate reader. With the help of calibration curve obtained by standard samples, we estimated the concentration of CYFRA-21-1 in processed saliva of OC patients.

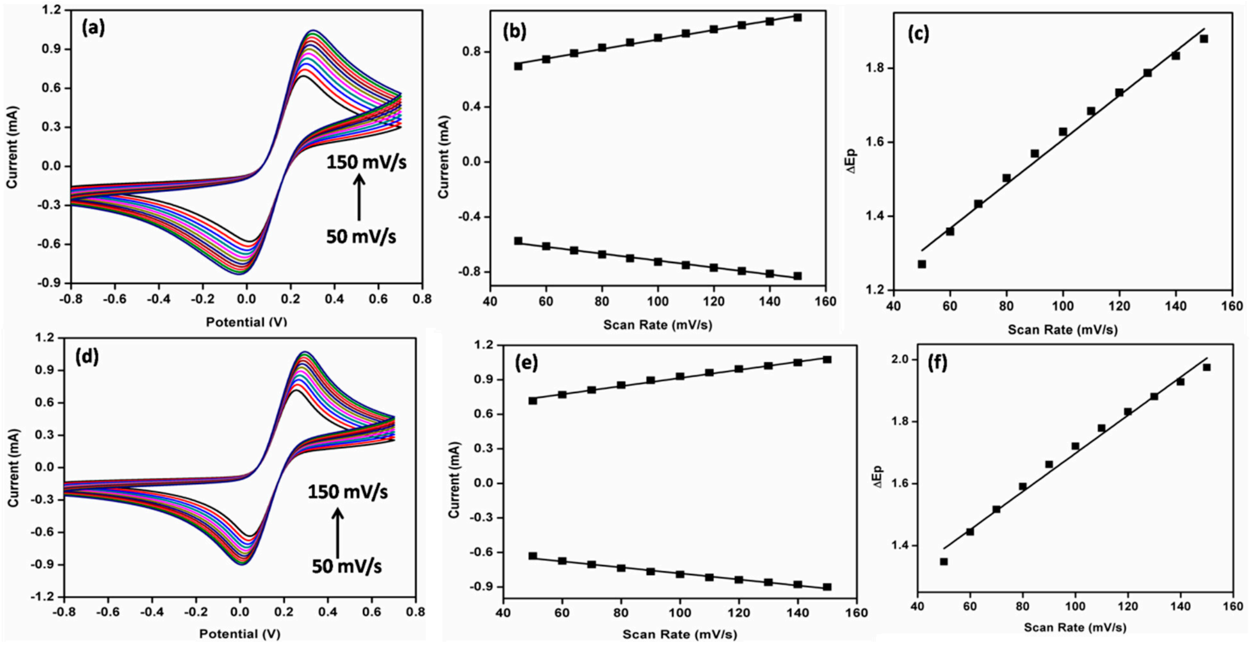

Figure S1: (a) CV response of APTES/nY_2O_3/ITO electrode as a function of scan rate (50-150 mV/s), (b) magnitude of anodic (I_{pa}) and cathodic (I_{pc}) peak current as a function of scan rate (mV/s) (c) difference potential $\Delta E_{p} = E_{pa}-E_{pc}$ as function of scan rate, (d) CV response of BSA/anti-CYFRA-21-1/APTES/nY_2O_3/ITO electrode as a function of scan rate (50-150 mV/s), (e) magnitude of anodic (I_{pa}) and cathodic (I_{pc}) peak current as a function of scan rate (mV/s) and (f) difference potential $\Delta E_{p} = E_{pa}-E_{pc}$ as function of scan rate

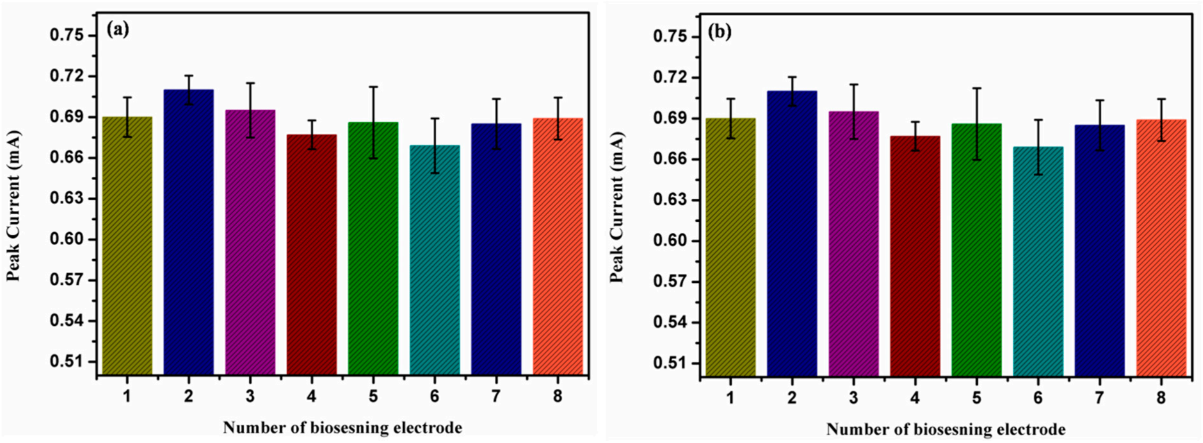

Figure S2: Reproducibility studies of BSA/anti-CYFRA-21-1/APTES/nY_2O_3/ITO immunoelectrode by using (a) CV and (b) EIS studies.

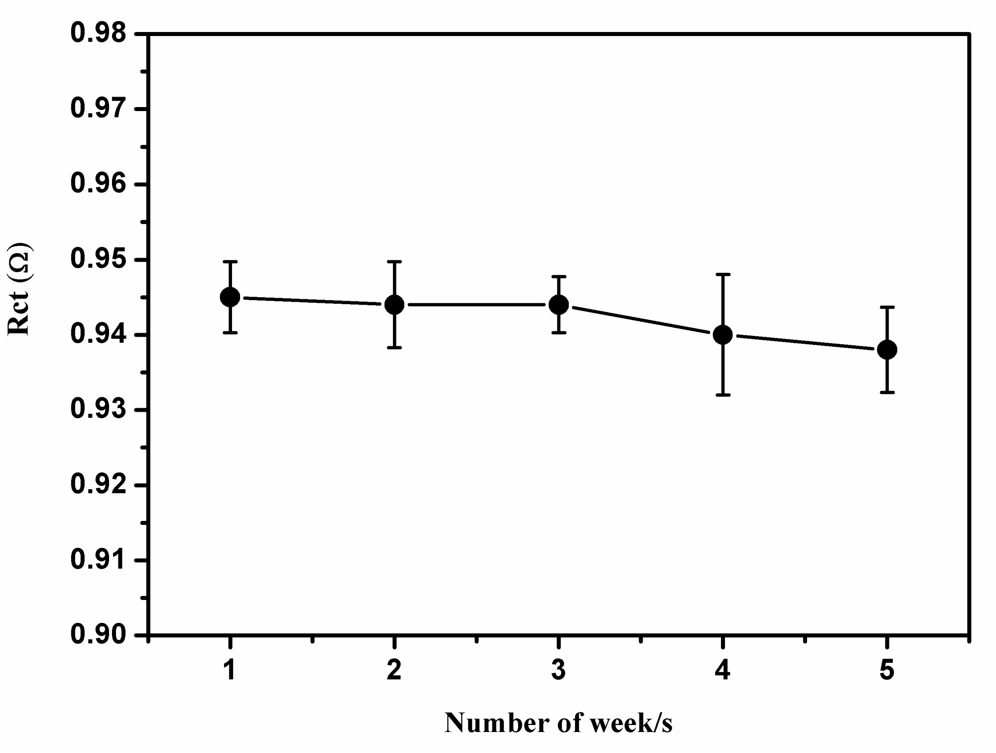

Figure S3: Shelf life studies of BSA/anti-CYFRA-21-1/APTES/nY_2O_3/ITO immunoelectrode via EIS studies.

Table S1: % Cell viability of HEK 295 Cells with respect to various concentration of nY_2O_3 along with % cell viability error bar (n=3).

S. No.	Concentration of nY_2O_3 ($\mu g\ mL^{-1}$)	% Cell viability (Mean value)	% Cell viability Error bar (±)
1.	0	100.00	0.91
2.	2	99.05	0.95
3.	5	98.80	1.94
4.	10	97.20	1.16
5.	20	96.89	1.45
6.	30	96.51	2.83
7.	50	95.32	2.28
8.	100	94.86	1.74

Table S2: Determination of CYFRA-21-1 concentration in saliva samples of oral cancer patient using BSA/anti-CYFRA-21-1/APTES/nY_2O_3/ITO immunoelectrodes.

S. No.	CYFRA-21-1 concentration determined using ELISA (in ng mL^{-1})	Rct value (KΩ) obtained for standard CYFRA-21-1 samples	Rct value (KΩ) obtained with in Patients saliva samples	% RSD
1.	13.35	1.838	1.955	4.36 %
2.	14.15	1.848	1.987	5.13 %
3.	12.10	1.833	1.920	3.28 %
4.	12.50	1.838	1.945	4.00 %
5.	15.55	1.857	2.013	5.70 %
6.	10.35	1.814	1.732	3.27 %
7.	11.65	1.830	1.786	1.72 %
